# Supplementary material for: β2-Adrenergic Signalling Promotes Cell Migration by Upregulating Expression of the Metastasis-Associated Molecule LYPD3
Source: Biology (Basel). 2020 Feb 22;9(2):39. doi: 10.3390/biology9020039 (PMC7168268; doi:10.3390/biology9020039)
Supplement: Supplementary file 1 [file biology-09-00039-s001.zip › Supplementary Table 1 Biology.docx]

| **Supplementary Table 1(A):** Enriched GO biological processes for MDA-MB-468 cells following norepinephrine stimulation. | |
| --- | --- |
| GO Biological Process | Genes |
| GO:0008283 Cell proliferation | KHDRBS1, SERPINF1, NRDC, TGFB2, TACC2 |
| GO:0016477 Cell migration | RHOA, SDC4, NRDC, TGFB2 |
| GO:0098609 Cell-cell adhesion | EEF1G, NDRG1, HSPA5 |
| GO:0007267 Cell-cell signalling | ADM, S100A9, TGFB2 |
| GO:0031532 Actin cytoskeleton reorganisation | S100A9, RHOA |
| GO:0044319 Wound healing, spreading of cells | RHOA, RHOC |

| **Supplementary Table 1(B):** Enriched GO biological processes for MDA-MB-231 cells following norepinephrine stimulation. | |
| --- | --- |
| GO Biological Process | Genes |
| GO:0043066 Negative regulation of apoptotic process | CDK1, HSP90B1, HSPA5, ANXA4, GAS6, PARK7, TMF1, TIMP1 |
| GO:0098609 Cell-cell adhesion | YWHAB, KTN1, HSPA5, SFN, PARK7 |
| GO:0008283 Cell proliferation | CDK1, TXNRD1, GAS6, TGFB2 |
| GO:0016477 Cell migration | CDK1, GAS6, TGFB2 |
| GO:0061024 Membrane organisation | YWHAB, SFN |
