# Supplementary material for: β2-Adrenergic Signalling Promotes Cell Migration by Upregulating Expression of the Metastasis-Associated Molecule LYPD3
Source: Biology (Basel). 2020 Feb 22;9(2):39. doi: 10.3390/biology9020039 (PMC7168268; doi:10.3390/biology9020039)
Supplement: Supplementary file 1 [file biology-09-00039-s001.zip › Supplementary Table 2 Biology.docx]

| **Supplementary Table 2**: Primer sequences used for qRT‑PCR. | | | |
| --- | --- | --- | --- |
| Primer Name | Forward Primer (5’-3’) | Reverse Primer (5’-3’) | Annealing Temperature (°C) |
| ADRα1A | TTTCTTAGTCATGCCCATTG | ATGTTTGGAAGACTGCTTC | 58 |
| ADRα1B | ATATAGTGGCCAAGAGAACC | TCGTGAAAGTTCTTGGAATG | 57 |
| ADRα1D | ACAAGCCTGCTGTATTTATC | TTGAGGGGAAGTAATAAGGG | 56 |
| ADRα2A | ACATGTTGCTAATGACAGTG | AGATAACAGACAAGAGGACC | 55 |
| ADRα2B | GAGACCCCTGAAGATACTG | CTCCTCTTCCTCCTCTTC | 58 |
| ADRα2C | AGTTCTTCTTCTGGATCGG | GAAGAGGATGTGCTTAAAGG | 56 |
| ADRβ1 | AAAAGGAAAGTTTGGGAAGG | CTCAGAGAGTGTAAAAACC | 56 |
| ADRβ2 | CACTCCTCTTATTTGCTCAC | AAACTTTAGACTTTGCTCGG | 56 |
| ADRβ3 | ATGAGACCTTAGTGTTCTCC | CATTCCATGGCTAAAGTGAG | 55 |
| LYPD3 | AGGTAATGAGAGTGCATACC | GACACAGTCACATTAGCTG | 55 |
| TBP | TATAATCCCAAGCGGTTTGC | GCTGGAAAACCCAACTTCTG | 57 |
| β-actin | CTCTTCCAGCCTTCCTTCCT | AGCACTGTGTTGGCGTACAG | 61 |
