# Supplementary material for: β2-Adrenergic Signalling Promotes Cell Migration by Upregulating Expression of the Metastasis-Associated Molecule LYPD3
Source: Biology (Basel). 2020 Feb 22;9(2):39. doi: 10.3390/biology9020039 (PMC7168268; doi:10.3390/biology9020039)
Supplement: Supplementary file 1 [file biology-09-00039-s001.zip › Supplementary Table 3 Biology.docx]

| **Supplementary Table 3**: Antibodies used in flow cytometry and immunohistochemistry assays. | | | | | |
| --- | --- | --- | --- | --- | --- |
| Antibody name | Species | Clone | Supplier | Product number | Concentration used |
| anti-human ADRα1D | mouse |  | Antibodies-Online, USA | ABIN2614848 | 2 μg/mL |
| anti-human ADRα2C | rabbit |  | Antibodies-Online, USA | ABIN1394816 | 4 μg/mL |
| anti-human ADRα1B | mouse | 9H45 | Antibodies-Online, USA | ABIN2196931 | 2 μg/mL |
| anti‑human ADRβ2 | mouse | RH11E1 | BIORAD, UK | MCA2784 | 4 μg/mL (FACS)  1:1000 (IF) |
| anti‑LYPD3 | rabbit |  | Abcam, UK | Ab151709 | 1:100 (IHC) |
