# Supplementary figures and images for: β2-Adrenergic Signalling Promotes Cell Migration by Upregulating Expression of the Metastasis-Associated Molecule LYPD3
Source: Biology (Basel). 2020 Feb 22;9(2):39. doi: 10.3390/biology9020039 (PMC7168268; doi:10.3390/biology9020039)

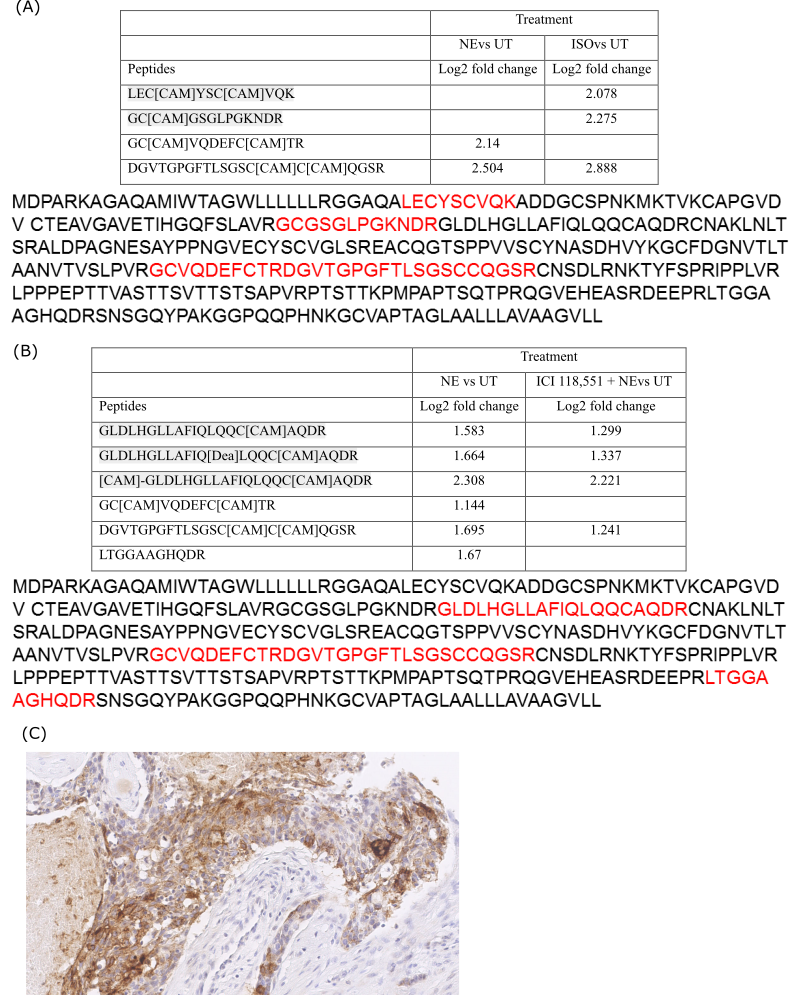

Supplement: Supplementary file 1 [file biology-09-00039-s001.zip › Supplementary Figure 4 Biology.docx]
